# Racial difference in mortality among COVID-19 hospitalizations in California

**DOI:** 10.1038/s41598-023-47124-6

**Published:** 2023-12-04

**Authors:** Muni Rubens, Venkataraghavan Ramamoorthy, Anshul Saxena, Juan Carlos Zevallos, Juan Gabriel Ruiz Pelaez, Md Ashfaq Ahmed, Zhenwei Zhang, Peter McGranaghan, Sandra Chaparro, Javier Jimenez

**Affiliations:** 1 Miami Cancer Institute, Baptist Health South Florida, Miami, FL USA; 2 Universidad Espíritu Santo, Samborondón, Ecuador; 3 Center for Advanced Analytics, Baptist Health South Florida, Miami, FL USA; 4 Miami Cardiac & Vascular Institute, Baptist Health South Florida, Miami, FL USA; 5 Herbert Wertheim College of Medicine, Florida International University, Miami, FL USA; 6 Charité-Universitätsmedizin Berlin, Augustenburger Platz 1, 10117 Berlin, Germany; 7 Advance Heart Failure and Pulmonary Hypertension, South Miami Hospital, Baptist Health South, Miami, FL USA; 8 Herbert Wertheim College of Medicine, Florida International University, Miami, FL USA; 9 Department of Internal Medicine and Cardiology, Charité-Universitätsmedizin Berlin, Berlin, Germany; 10 Freie Universität Berlin and Humboldt Universität Zu Berlin, Augustenburger Platz 1, 10117 Berlin, Germany

**Keywords:** Infectious diseases, Health care, Signs and symptoms

## Abstract

In the US, racial disparities in hospital outcomes are well documented. We explored whether race was associated with all-cause in-hospital mortality and intensive care unit (ICU) admission among COVID-19 patients in California. This was a retrospective analysis of California State Inpatient Database during 2020. Hospitalizations ≥ 18 years of age for COVID-19 were included. Cox proportional hazards with mixed effects were used for associations between race and in-hospital mortality. Logistic regression was used for the association between race and ICU admission. Among 87,934 COVID-19 hospitalizations, majority were Hispanics (56.5%), followed by White (27.3%), Asian, Pacific Islander, Native American (9.9%), and Black (6.3%). Cox regression showed higher mortality risk among Hispanics, compared to Whites (hazard ratio, 0.91; 95% CI 0.87–0.96), Blacks (hazard ratio, 0.87; 95% CI 0.79–0.94), and Asian, Pacific Islander, Native American (hazard ratio, 0.89; 95% CI 0.83–0.95). Logistic regression showed that the odds of ICU admission were significantly higher among Hispanics, compared to Whites (OR, 1.70; 95% CI 1.67–1.74), Blacks (OR, 1.70; 95% CI 1.64–1.78), and Asian, Pacific Islander, Native American (OR, 1.82; 95% CI 1.76–1.89). We found significant disparities in mortality among COVID-19 hospitalizations in California. Hispanics were the worst affected with the highest mortality and ICU admission rates.

## Introduction

The US has the greatest number of COVID-19 cases and highest mortality rates in the world. Recent estimates show that there were 84 million cases and 1 million deaths accountable to COVID-19 in the US^1^. Ever since the beginning of the pandemic, studies have shown that there were racial disparities with respect to morbidity and mortality associated with COVID-19^2–4^. For example, COVID-19 related mortality was disproportionately higher in Midwestern Latino counties as well as counties with greater number of people speaking Spanish as their first language^4^. Similarly, a study done in Louisiana showed that though Blacks represented only 30% of total population, 77% of COVID-19 hospitalizations and 70% COVID-19 related deaths were among Blacks, indicating existing racial disparities^5^. Understanding and overcoming existing racial disparities in COVID-19 such as comorbidity burden, consulting patterns and health care access, health seeking behavior, and intergenerational interaction are considered extremely important public health priority and challenge^6^. Availability of information on the extent and type of racial disparities could help in identifying the factors associated with these inequalities and measures to overcome them in order to achieve greater control over COVID-19. The existence of greater number of cases and mortality among racial minorities could lead to lower control over the spread of the virus. Achieving and ensuring equity across different population groups is especially important for preventing and treating COVID-19. In this study we sought to explore whether there were disparities in COVID-19 related in-hospital mortality based on race in the state of California. The State Inpatient Database (SID) of California provides a large database with pertinent information on COVID-19 hospitalizations such as demographics, clinical risk profile, and mortality. California also ranks the highest within the US in terms of total number of CODVID-19 cases as well as mortality^1^.

## Methods

### Study design and data source

This study was a retrospective analysis of the California SID collected during 2020. The Agency for Healthcare Research and Quality (AHRQ) developed SID to collect clinical data from patients admitted to participating hospitals within the state of Califirnia^7^. Annually the SID gathers discharge data from more than 90% of the patients who are admitted to more than 97% of community hospitals, and the data does not differ significantly from non-participating hospitals. The Strengthening the Reporting of Observational Studies in Epidemiology (STROBE) guideline was used for ensuring the quality of our study^8^. This study was performed in accordance with the guidelines of the Declaration of Helsinki. The study has been approved by the Institutional Review Board (IRB) of Florida International University (IRB-22-0473-NHSR). The requirement for informed consent was waived by the Florida International University IRB due to the retrospective nature of the study.

### Study population

All hospitalizations with age 18 years and above and having COVID-19 as primary diagnosis were included for the analysis. These hospitalizations were subsequently stratified by racial groups (Whites, Blacks, Hispanics, and Asian, Pacific Islander, Native American). The International Classification of Diseases, Tenth Revision, Clinical Modification (ICD-10-CM) diagnosis and procedure codes were used for retrieving hospitalizations and procedures. COVID-19 hospitalizations were identified using ICD-10-CM diagnosis code U07.1.

### Study variables and outcomes

The primary outcome of the study was in-hospital mortality, and secondary outcome was ICU admission. Comorbidities included conditions such as hypertension, diabetes mellitus, hyperlipidemia, obesity, atrial fibrillation, coagulation disorder, peripheral vascular disease, liver disease, chronic renal failure, tobacco use, alcohol abuse, drug abuse, stroke, congestive heart failure (CHF), chronic pulmonary disease, metastatic cancer, and anemia. We also included Elixhauser comorbidity index as a composite measure of all comorbidities. These variables were identified using ICD-10-CM diagnosis and procedure codes.

### Statistical analysis

The distribution of demographics and clinical characteristics of COVID-19 hospitalizations was stratified by race. These variables were described as frequencies and percentages. Survival analysis was conducted using Kaplan–Meier estimator and compared differences in COVID-19 related mortality across different racial groups. Cox proportional hazard regression analyses were used to compare COVID-19 related mortality across different races. The regression models were adjusted for covariates such as age, sex, insurance, hypertension, diabetes mellitus, hyperlipidemia, obesity, atrial fibrillation, coagulation disorder, peripheral vascular disease, liver disease, chronic renal failure, tobacco use, alcohol abuse, drug abuse, stroke, CHF, chronic pulmonary disease, metastatic cancer, and anemia. Finally, we conducted binary logistic regression to see whether ICU admission differed among different racial groups after adjusting for the covariates. In the regression models, we also looked for the interaction of race with factors such as age, sex, diabetes, obesity, and hypertension. Statistical significance was set at *P* < 0.05 and all tests were two sided. All statistical analyses were conducted using SAS, version 9.4 (SAS Inc., Cary, NC).

## Results

A total of 87,934 COVID-19 hospitalizations were included for the analysis. The majority of them were Hispanics (56.5%), followed by Whites (27.3%), Asian, Pacific Islander, Native American (9.9%), and Blacks (6.3%). The age distribution of the sample showed that the majority were between the ages 45–85 years. Among Hispanic hospitalizations, significant majority (43.9%) were in the age group 45–64 years, while among Whites (46.5%) and Asian, Pacific Islander, Native American (40.0%), majority were in the age group 65–84 years. There were greater proportions of male hospitalizations in the total sample as well as in all racial groups. The majority of the patients in the total sample had Medicare coverage (43.4%). Hispanics differed from other races with the respect to insurance coverage since the majority of them had Medicaid, whereas the majority in other races had Medicare coverage. The most common comorbidities observed in the total sample were hypertension (61.6%), hyperlipidemia (39.3%), obesity (28.1%), chronic renal failure (19.3%), and chronic pulmonary disease (18.9%). The rates of atrial fibrillation, coagulation disorder, and peripheral vascular disease were highest among Whites, whereas the rates of hypertension, hyperlipidemia, chronic renal failure, tobacco use, alcohol abuse, drug abuse, stroke, congestive heart failure, chronic pulmonary disease, metastatic cancer, and anemia were highest among Blacks. The rates of diabetes mellitus, obesity, and liver disease were highest among Hispanics. Elixhauser comorbidity index ≥ 3 was observed among 59.4% of total sample and was substantially higher among Blacks (73.6%), followed by Whites (68.4%), Asian, Pacific Islander, Native American (59.2%), and Hispanics (53.5%). All demographic and clinical characteristics showed significant differences between racial groups (Table 1).

**Table 1:** Demographic and clinical characteristics of hospitalized characteristics of COVID-19 hospitalizations by race in California.

Characteristic	Total N = 87,934	White n = 23,972 (27.3%)	Black n = 5513 (6.3%)	Hispanic n = 49,719 (56.5%)	Asian, Pacific Islander, Native American n = 8730 (9.9%)	*P* value
Age, n (%)						< 0.001
18–44 years	14,064 (16.0%)	1731 (7.2%)	767 (13.9%)	10,564 (21.2%)	1002 (11.5%)	
45–64 years	33,611 (38.2%)	6567 (27.4%)	2151 (39.0%)	21,823 (43.9%)	3070 (35.2%)	
65–84 years	31,391 (35.7%)	11,142 (46.5%)	2148 (39.0%)	14,608 (29.4%)	3493 (40.0%)	
≥ 85 years	8868 (10.1%)	4532 (18.9%)	447 (8.1%)	2724 (5.5%)	1165 (13.3%)	
Sex, n (%)						< 0.001
Male	48,315 (54.9%)	12,840 (53.6%)	2922 (53.0%)	27,965 (56.2%)	4588 (52.6%)	
Female	39,617 (45.1%)	11,132 (46.4%)	2591 (47.0%)	21,753 (43.8%)	4141 (47.4%)	
Insurance, n (%)						< 0.001
Medicare	38,179 (43.4%)	15,394 (64.2%)	2746 (49.8%)	15,958 (32.1%)	4081 (46.8%)	
Medicaid	24,660 (28.1%)	2794 (11.7%)	1321 (24.0%)	19,082 (38.4%)	1463 (16.8%)	
Private insurance	20,602 (23.4%)	4935 (20.6%)	1110 (20.1%)	11,769 (23.7%)	2788 (31.9%)	
Uninsured	1588 (1.8%)	143 (0.6%)	73 (1.3%)	1244 (2.5%)	128 (1.5%)	
Other	2866 (3.3%)	699 (2.9%)	259 (4.7%)	1639 (3.3%)	269 (3.1%)	
Clinical risk profile, n (%)						
Hypertension	54,132 (61.6%)	16,366 (68.3%)	4126 (74.8%)	27,625 (55.6%)	6015 (68.9%)	< 0.001
Diabetes mellitus	14,820 (16.9%)	3044 (12.7%)	795 (14.4%)	9496 (19.1%)	1485 (17.0%)	0.009
Hyperlipidemia	34,561 (39.3%)	10,959 (45.7%)	2223 (40.3%)	17,106 (34.4%)	4273 (48.9%)	0.005
Obesity	24,728 (28.1%)	5926 (24.7%)	1702 (30.9%)	15,750 (31.7%)	1350 (15.5%)	< 0.001
Atrial fibrillation	8812 (10.0%)	4197 (17.5%)	541 (9.8%)	3105 (6.2%)	969 (11.1%)	< 0.001
Coagulation disorder	9976 (11.3%)	3117 (13.0%)	576 (10.4%)	5236 (10.5%)	1047 (12.0%)	0.008
Peripheral vascular disease	5928 (6.7%)	2531 (10.6%)	563 (10.2%)	2125 (4.3%)	709 (8.1%)	< 0.001
Liver disease	5287 (6.0%)	1115 (4.7%)	282 (5.1%)	3304 (6.6%)	586 (6.7%)	0.009
Chronic renal failure	16,933 (19.3%)	5414 (22.6%)	1742 (31.6%)	7819 (15.7%)	1958 (22.4%)	< 0.001
Tobacco use	4031 (4.6%)	1389 (5.8%)	510 (9.3%)	1823 (3.7%)	309 (3.5%)	< 0.001
Alcohol abuse	1985 (2.3%)	585 (2.4%)	136 (2.5%)	1195 (2.4%)	69 (0.8%)	< 0.001
Drug abuse	2527 (2.9%)	886 (3.7%)	362 (6.6%)	1185 (2.4%)	94 (1.1%)	< 0.001
Stroke	3449 (3.9%)	1125 (4.7%)	399 (7.2%)	1469 (3.0%)	456 (5.2%)	0.019
Congestive heart failure	11,704 (13.3%)	4602 (19.2%)	1206 (21.9%)	4846 (9.7%)	1050 (12.0%)	< 0.001
Chronic pulmonary disease	16,619 (18.9%)	6847 (28.6%)	1642 (29.8%)	6571 (13.2%)	1559 (17.9%)	0.008
Metastatic cancer	699 (0.8%)	267 (1.1%)	69 (1.3%)	284 (0.6%)	79 (0.9%)	0.008
Anemia	2738 (3.1%)	765 (3.2%)	239 (4.3%)	1438 (2.9%)	296 (3.4%)	0.019
Elixhauser comorbidity index, n (%)						< 0.001
0	6228 (7.1%)	993 (4.1%)	161 (2.9%)	4484 (9.0%)	590 (6.8%)	
1 or 2	29,485 (33.5%)	6593 (27.5%)	1292 (23.4%)	18,629 (37.5%)	2971 (34.0%)	
≥ 3	52,221 (59.4%)	16,386 (68.4%)	4060 (73.6%)	26,606 (53.5%)	5169 (59.2%)	
Mortality, n (%)	10,065 (11.5%)	3087 (12.9%)	576 (10.5%)	5358 (10.8%)	1044 (12.0%)	< 0.001

**Figure 1:**
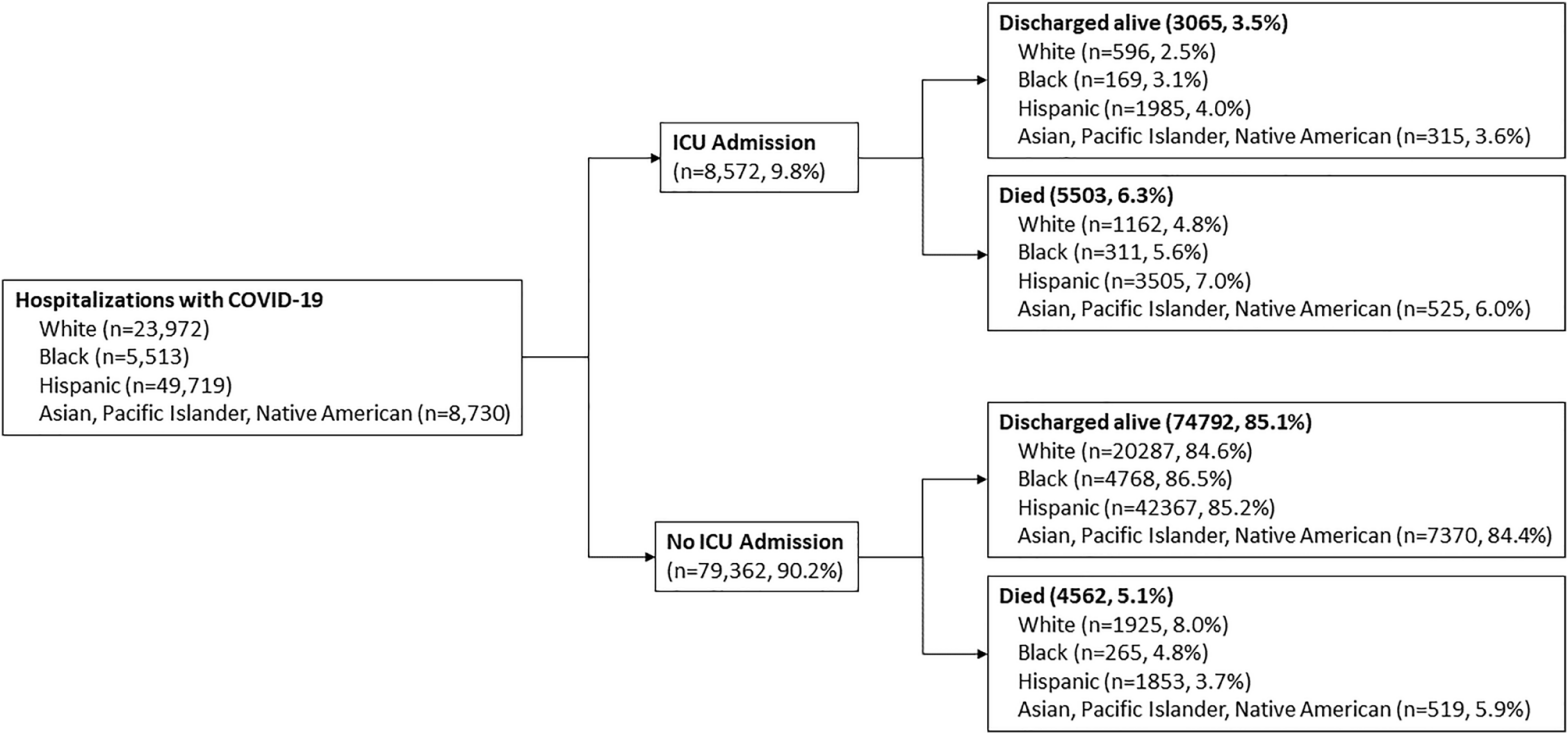
ICU admission and in-hospital mortality among COVID-19 hospitalizations (N = 87,934).

Among total hospitalizations, all-cause, in-hospital mortality was 11.5%. Of these hospitalizations, 9.8% had ICU admission, whereas 90.2% did not. Among hospitalizations with ICU admission, 35.8% were discharged alive whereas 64.2% died. Among hospitalizations with ICU admission, mortality rates were highest among Hispanics (63.6%), followed by Whites (21.1%), Asian, Pacific Islander, Native American (9.5%), and Blacks (5.6%). Among hospitalizations without ICU admission, 94.2% were discharged alive whereas 5.8% died. Among hospitalizations without ICU admission, mortality rates were highest among Whites (42.1%), followed by Hispanics (40.6%), Asian, Pacific Islander, Native American (11.3%), and Blacks (5.8%). Figure 1 shows ICU admission and in-hospital mortality among COVID-19 hospitalizations.

**Figure 2:**
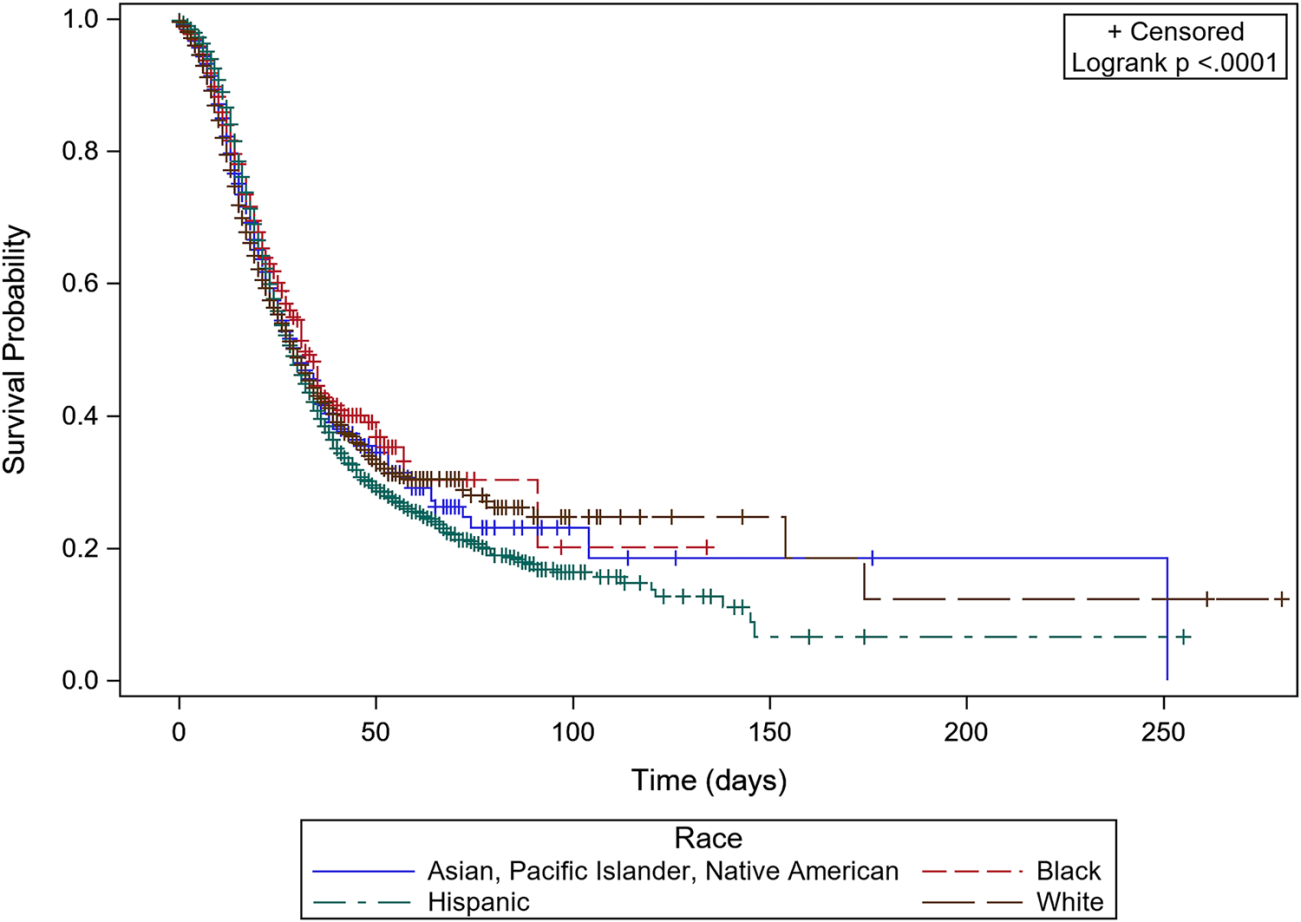
Kaplan Meier curves showing in-hospital mortality rates among COVID-19 hospitalizations stratified by race.

Survival analysis stratified by race showed that Hispanics had the lowest survival rates, followed by Asian, Pacific Islander, Native American, Whites, and Blacks. Survival rates differed significantly between the races (Logrank *P* < 0.001). Figure 2 shows Kaplan Meier curves for in-hospital mortality among COVID-19 hospitalizations stratified by race.

Cox proportional regression analysis showed that the risk of mortality was significantly higher among Hispanics, compared to Whites (HR, 0.91; 95% CI 0.87–0.96), Blacks (HR, 0.87; 95% CI 0.79–0.94), and Asian, Pacific Islander, Native American (HR, 0.89; 95% CI 0.83–0.95). The risk of mortality was also significantly higher across increasing age groups and among men (HR, 1.16; 95% CI 1.11–1.21). The comorbidities associated with greater risk of mortality were diabetes mellitus (HR, 1.20; 95% CI 1.13–1.27), obesity (HR, 1.07; 95% CI 1.02–1.12), atrial fibrillation HR, 1.38; 95% CI 1.31–1.44), coagulation disorder (HR, 1.15; 95% CI 1.10–1.20), liver disease (HR, 1.37; 95% CI 1.28–1.47), chronic renal failure (HR, 1.36; 95% CI 1.29–1.42), congestive heart failure (HR, 1.23; 95% CI 1.17–1.29), and metastatic cancer (HR, 1.59; 95% CI 1.36–1.87). Table 2 shows hazard ratios for in-hospital mortality among COVID-19 hospitalizations. Cox proportional regression analysis with interaction between race and factors such as age, sex, diabetes, obesity, and hypertension did not show any significant interactions (Supplementary Table 1).

**Table 2:** Hazard ratios for in-hospital mortality among COVID-19 hospitalizations in California.

Characteristic	Hazard ratio (95% CI)	*P* value
Race		
Hispanic	References	
White	0.91 (0.87–0.96)	< 0.001
Black	0.87 (0.79–0.94)	< 0.001
Asian, Pacific Islander, Native American	0.89 (0.83–0.95)	< 0.001
Age		
18–44 years	References	
45–64 years	1.70 (1.52–1.91)	< 0.001
65–84 years	2.78 (2.46–3.13)	< 0.001
≥ 85 years	5.37 (4.72–6.10)	< 0.001
Sex		
Female	References	
Male	1.16 (1.11–1.21)	< 0.001
Insurance		
Medicare	References	
Medicaid	0.89 (0.83–0.95)	< 0.001
Private insurance	0.82 (0.76–0.88)	< 0.001
Uninsured	1.00 (0.81–1.23)	0.987
Other	0.80 (0.70–0.92)	0.001
Hypertension	1.03 (0.98–1.08)	0.317
Diabetes mellitus	1.20 (1.13–1.27)	< .0001
Hyperlipidemia	0.99 (0.95–1.03)	0.671
Obesity	1.07 (1.02–1.12)	0.009
Atrial fibrillation	1.38 (1.31–1.44)	< 0.001
Coagulation disorder	1.15 (1.10–1.20)	< 0.001
Peripheral vascular disease	1.01 (0.95–1.08)	0.717
Liver disease	1.37 (1.28–1.47)	< 0.001
Chronic renal failure	1.36 (1.29–1.42)	< 0.001
Tobacco use	1.01 (0.90–1.12)	0.922
Alcohol abuse	1.05 (0.92–1.20)	0.436
Drug abuse	0.89 (0.77–1.04)	0.138
Stroke	1.05 (0.98–1.13)	0.182
Congestive heart failure	1.23 (1.17–1.29)	< 0.001
Chronic pulmonary disease	0.96 (0.91–1.00)	0.069
Metastatic cancer	1.59 (1.36–1.87)	< 0.001
Anemia	0.86 (0.77–1.15)	0.103

Comparison of ICU admission rates among racial groups showed that, ICU admission was highest among Hispanics (n = 5494, 11.1%), followed by Asian, Pacific Islander, Native American (n = 840, 9.6%), Blacks (n = 480, 8.7%), and Whites (1758, 7.3%). Logistic regression analysis showed that the odds of ICU admission were significantly higher among Hispanics, compared to Whites (OR, 1.70; 95% CI 1.67–1.74), Blacks (OR, 1.70; 95% CI 1.64–1.78), and Asian, Pacific Islander, Native American (OR, 1.82; 95% CI 1.76–1.89). Table 3 shows the full model. Logistic regression analysis with interaction between race and factors such as age, sex, diabetes, obesity, and hypertension did not show any significant interactions (Supplementary Table 2).

**Table 3:** Factors associated with ICU admission among COVID-19 hospitalizations in California.

Characteristic	Hazard ratio (95% CI)	*P* value
Race		
Hispanic	References	
White	1.70 (1.67–1.74)	< 0.001
Black	1.70 (1.64–1.78)	< 0.001
Asian, Pacific Islander, Native American	1.82 (1.76–1.89)	< 0.001
Age		
18–44 years	References	
45–64 years	2.85 (2.53–3.20)	< 0.001
65–84 years	6.11 (5.39–6.93)	< 0.001
≥ 85 years	11.11 (9.69–12.73)	< 0.001
Sex		
Female	References	
Male	0.71 (0.68–0.74)	< 0.001
Insurance		
Medicare	References	
Medicaid	1.02 (0.95–1.09)	0.089
Private insurance	0.81 (0.74–0.87)	0.002
Uninsured	0.79 (0.64–0.99)	0.014
Other	0.91 (0.79–1.06)	0.831
Hypertension	1.06 (1.00–1.12)	0.052
Diabetes mellitus	1.10 (1.03–1.17)	0.002
Hyperlipidemia	0.90 (0.86–1.95)	0.071
Obesity	1.32 (1.25–1.39)	< 0.001
Atrial fibrillation	1.96 (1.85–2.07)	< 0.001
Coagulation disorder	2.29 (2.17–2.42)	< 0.001
Peripheral vascular disease	0.82 (0.76–1.89)	0.078
Liver disease	1.79 (1.65–1.94)	< 0.001
Chronic renal failure	1.54 (1.46–1.62)	< 0.001
Tobacco use	0.87 (0.77–1.98)	0.084
Alcohol abuse	0.95 (0.82–1.10)	0.510
Drug abuse	0.85 (0.72–1.03)	0.066
Stroke	1.52 (1.39–1.66)	< 0.001
Congestive heart failure	1.40 (1.33–1.49)	< 0.001
Chronic pulmonary disease	1.06 (1.02–1.12)	0.037
Metastatic cancer	1.82 (1.50–2.21)	< 0.001
Anemia	1.00 (0.89–1.13)	0.944

## Discussion

In our cohort of 87,934 COVID-19 hospitalizations in California, Hispanics had significantly higher all-cause, in-hospital mortality and significantly lower ICU admission rates, compared to other races. All-cause, in-hospital mortality was 11.5% in the total sample, whereas it was 64.2% among hospitalizations with ICU admission, and 5.8% among hospitalizations without ICU admission. Similar to previous studies, mortality rates were significantly higher among older adults and men.

Similar to our findings, a cross-sectional study using publicly reported COVID-19 mortality data showed that Hispanics had 2.17 times greater risk for mortality, compared to Whites in California^9^. In addition, this study also showed that mortality rates were significantly higher among Hispanics in 13 states including Missouri (5.26), New York (3.98), and Massachusetts (2.60). Nevertheless, several other studies showed that mortality rates due to COVID-19 did not differ significantly between Hispanics and other races^10, 11^. For example, in a retrospective cohort study that included 4,413 patients mortality rates did not differ significantly between Blacks and Hispanics^10^. Similarly, in a multicenter, retrospective study of laboratory-confirmed COVID-19 patients aged ≥ 18 years in-hospital mortality did not differ between Whites, Blacks, and Hispanics^11^. In spite of these findings, it should be noted that these studies are either single institution studies conducted outside California or national studies within the United States. Hence, our study findings, which show that mortality rates were higher among Hispanics could be due to the characteristics specific to Hispanics in California. The racial characteristics of Californian population shows predominance of Hispanics (39.4%), followed by Whites (36.5%), Asian (15.5%), and Blacks (6.5%)^12^. Although Hispanics constitute the majority in California, they are considered a disadvantaged group with regards to many economic and social factors which could increase the impact of COVID-19 in this population. For example, Hispanics in California are 8 times more likely to reside in areas with greater risk of exposure to COVID-19, compared to Whites^13^. In addition, there are significantly greater number of cumulative COVID-19 cases and lower rates of cumulative testing among Hispanics, compared to other races in California^13^.

In our study, COVID-19 mortality rates were highest among Whites (12.9%), followed by Asian, Pacific Islander, Native American (12.0%), Hispanics (10.8%), and Blacks (10.5%). The highest mortality rates among Whites could be due to the higher comorbidity burden measured by the Elixhauser comorbidity index. However, after adjusting for covariates, our multivariable analysis showed that the risk for mortality was the highest for Hispanics, compared to other races. The distribution of comorbidities showed that three of them were highest among Whites and Hispanics each, whereas eleven of them were highest among Blacks. Notably, the comorbidities highest among Hispanics were diabetes mellitus, obesity, and liver disease. Many previous studies have shown that diabetes and obesity are independent risk factors for mortality among COVID-19 patients^14, 15^. Subsequently, further analysis within our study also showed that higher mortality among Hispanics was due to the higher prevalence of these comorbidities. In addition to diabetes and obesity, covariates such as atrial fibrillation, coagulation disorder, liver disease, chronic renal failure, congestive heart failure, and metastatic cancer were independent risk factor for mortality in the total sample. Previous studies have shown that preexisting comorbidities could adversely affect the prognosis and survival of COVID-19 patients^16–18^. Given the greater proportion of comorbidities among the Black population in our study, it is surprising that Hispanics had greater mortality risk than Blacks. The contribution of each comorbidity towards mortality should be researched in greater detail. In our study, Hispanics had greater rates of diabetes, obesity, and liver disease which are known risk factors for mortality in COVID-19. Although Blacks had a greater number of comorbidities, most of them were not independent risk factors for mortality. In addition, we could not assess the stage and severity of the disease when the patients were admitted to the hospital which could be different among different races. Also, we could include only the available comorbidities in our study. There could be other comorbidities that were not available in the database, which could have contributed to the racial disparities in mortality observed in our study. This could have led to bias in our estimates; however, we included the available comorbidities in our models to reduce this bias. Future studies should focus on these factors while researching racial disparities in COVD-19 outcomes.

In our study ICU admission rates were higher Hispanic patients, compared to all other racial groups. Similar to our findings, a study done by Lee found that Hispanics and other minorities had higher in-hospital mortality and higher ICU admission rates, compared to Whites^19^. Similarly, another cross-sectional study found that Hispanics, Blacks, and Asian, Pacific Islander, Native American had greater rates of COVID-19 hospitalization, ICU admission, and in-hospital mortality than Whites^20^. This shows that Hispanic populations are more vulnerable and susceptible to adverse COVID-19 outcomes and received greater access to care and advanced treatments.

We also found that mortality was higher among male hospitalizations and increased with increasing age, which was consistent with findings in previous studies. For example, a meta-analysis by Biswasa and colleagues showed that COVID-19 related mortality was significantly higher among male patients and increased with increasing age^21^. Increased COVID-19 mortality among males could be due be explained by the role of male hormones towards increased expression of angiotensin-converting enzyme 2 (ACE-2) making them more vulnerable to SARS-CoV-2 infection and adverse prognosis as well^22^. In addition, ACE-2 expression is achieved by activation of ACE-2 gene on X chromosome^23^. Females having two copies of X chromosome could be heterozygous while males are always homozygous, making them more susceptible to greater levels of ACE-2 expression^23^. Females may have greater protection against adverse clinical outcomes due to mosaicism because of X-linked heterozygous alleles^23^. Greater COVID-19 related mortality among older adults could be due to factors such as increased expression of ACE-2, decreased immunity, decreased organ function, depleting physiologic reserves and vitality, greater levels of preexisting comorbidities^24, 25^.

## Limitations

In spite of our findings, our study had some limitations. ICD-10 codes were used for identifying hospitalizations with COVID-19 and preexisting comorbidities. This could have resulted in coding errors and misclassification bias. SID being an administrative database does not have laboratory and treatment related data. Unavailability of laboratory data restricted us from stratifying hospitalizations based on severity of the disease. This could have affected the accuracy of our findings. Unavailability of treatment related data including medications such as remdesivir, tocilizumab, and steroids could have affected the results of our study because they are important cofounding factors affecting COVID-19 related mortality. Since we used hospitalization data, we could not get data prior to admission and post-discharge. Mortality rates due to COVID-19 could be different if mortality prior to admission and post-discharge would be considered. The findings in our study could be due to the specific characteristics of Hispanic population within California. Therefore, our findings should be interpreted cautiously and not generalized to other Hispanic populations within the United Sates.

## Conclusion

In this study of COVID-19 hospitalizations in California, the risk of all-cause in-hospital mortality was significantly higher among Hispanics and associated with older age, male sex, and comorbidities. Given the existence of racial disparities in hospital outcomes among COVID-19 patients, the overarching objectives of providing equitable care, eliminating disparities, and achieving comparable outcomes remain challenges to be overcome. Further research should focus on identifying the exact mechanisms for the racial disparities in COVID-19 outcomes observed in our study. This would help in developing initiatives for overcoming these disparities and achieving equitable distribution of healthcare.

### Supplementary Information

Supplementary Tables.

## Data Availability

The datasets generated and analysed during the current study are available in the Healthcare Cost and Utilization Project (HCUP) State Inpatient Databases (SID) at: https://www.hcup-us.ahrq.gov/sidoverview.jsp.
