# Supplementary material for: Racial difference in mortality among COVID-19 hospitalizations in California
Source: Sci Rep. 2023 Dec 4;13:21378. doi: 10.1038/s41598-023-47124-6 (PMC10696031; doi:10.1038/s41598-023-47124-6)
Supplement: Supplementary file 1 — Supplementary Tables. [file 41598_2023_47124_MOESM1_ESM.doc]

Supplementary Table 1. Hazard ratios for in-hospital mortality among COVID-19 hospitalizations in California

| **Characteristic** | **Hazard ratio (95% CI)** | ***P* value** |
| --- | --- | --- |
| Race |  |  |
| Hispanic | References |  |
| White | 0.89 (0.83-0.97) | <0.001 |
| Black | 0.83 (0.74-0.98) | <0.001 |
| Asian, Pacific Islander, Native American | 0.80 (0.75-0.95) | <0.001 |
| Age |  |  |
| 18-44 years | References |  |
| 45-64 years | 1.68 (1.50-1.90) | <0.001 |
| 65-84 years | 2.72 (2.50-3.17) | <0.001 |
| ≥85 years | 5.42 (4.78-6.17) | <0.001 |
| Sex |  |  |
| Female | References |  |
| Male | 1.21 (1.09-1.25) | <0.001 |
| Insurance |  |  |
| Medicare | References |  |
| Medicaid | 0.85 (0.79-0.98) | <0.001 |
| Private insurance | 0.83 (0.74-0.89) | <0.001 |
| Uninsured | 1.02 (0.87-1.26) | 0.953 |
| Other | 0.83 (0.71-0.96) | 0.001 |
| Hypertension | 1.07 (0.95-1.10) | 0.346 |
| Diabetes mellitus | 1.23 (1.11-1.26) | <.0001 |
| Hyperlipidemia | 0.93 (0.89-1.06) | 0.623 |
| Obesity | 1.08 (1.01-1.16) | 0.018 |
| Atrial fibrillation | 1.42 (1.33-1.47) | <0.001 |
| Coagulation disorder | 1.16 (1.11-1.25) | <0.001 |
| Peripheral vascular disease | 1.03 (0.94-1.11) | 0.747 |
| Liver disease | 1.36 (1.23-1.49) | <0.001 |
| Chronic renal failure | 1.39 (1.31-1.45) | <0.001 |
| Tobacco use | 1.11 (0.96-1.14) | 0.969 |
| Alcohol abuse | 1.07 (0.94-1.23) | 0.412 |
| Drug abuse | 0.86 (0.75-1.07) | 0.167 |
| Stroke | 1.08 (0.94-1.15) | 0.112 |
| Congestive heart failure | 1.21 (1.16-1.27) | <0.001 |
| Chronic pulmonary disease | 0.95 (0.89-1.03) | 0.076 |
| Metastatic cancer | 1.62 (1.39-1.81) | <0.001 |
| Anemia | 0.81 (0.74-1.16) | 0.111 |
| Race*age | 1.21 (0.82-3.18) | 0.134 |
| Race*sex | 0.92 (0.83-1.15) | 0.169 |
| Race*diabetes | 0.96 (0.81-1.17) | 0.081 |
| Race*obesity | 0.91 (0.76-1.15) | 0.135 |
| Race*hypertension | 0.89 (0.78-1.04) | 0.092 |

Supplementary Table 2. Factors associated with ICU admission among COVID-19 hospitalizations in California.

| **Characteristic** | **Odds ratio (95% CI)** | ***P* value** |
| --- | --- | --- |
| Race |  |  |
| Hispanic | References |  |
| White | 1.72 (1.63-1.76) | <0.001 |
| Black | 1.73 (1.61-1.82) | <0.001 |
| Asian, Pacific Islander, Native American | 1.84 (1.75-1.92) | <0.001 |
| Age |  |  |
| 18-44 years | References |  |
| 45-64 years | 2.84 (2.57-3.23) | <0.001 |
| 65-84 years | 6.15 (5.42-6.99) | <0.001 |
| ≥85 years | 11.15 (9.72-12.81) | <0.001 |
| Sex |  |  |
| Female | References |  |
| Male | 0.78 (0.64-0.79) | <0.001 |
| Insurance |  |  |
| Medicare | References |  |
| Medicaid | 1.09 (0.94-1.13) | 0.047 |
| Private insurance | 0.86 (0.77-0.91) | 0.016 |
| Uninsured | 0.81 (0.62-0.98) | 0.023 |
| Other | 0.93 (0.77-1.09) | 0.846 |
| Hypertension | 1.05 (1.00-1.17) | 0.059 |
| Diabetes mellitus | 1.11 (1.02-1.19) | 0.004 |
| Hyperlipidemia | 0.92 (0.87-1.96) | 0.077 |
| Obesity | 1.34 (1.27-1.42) | <0.001 |
| Atrial fibrillation | 1.98 (1.83-2.10) | <0.001 |
| Coagulation disorder | 2.32 (2.19-2.48) | <0.001 |
| Peripheral vascular disease | 0.87 (0.74-1.94) | 0.082 |
| Liver disease | 1.81 (1.67-1.96) | <0.001 |
| Chronic renal failure | 1.58 (1.44-1.67) | <0.001 |
| Tobacco use | 0.89 (0.73-2.01) | 0.086 |
| Alcohol abuse | 0.97 (0.81-1.13) | 0.510 |
| Drug abuse | 0.86 (0.71-1.05) | 0.069 |
| Stroke | 1.56 (1.36-1.69) | <0.001 |
| Congestive heart failure | 1.43 (1.36-1.52) | <0.001 |
| Chronic pulmonary disease | 1.09 (1.03-1.17) | 0.041 |
| Metastatic cancer | 1.87 (1.52-2.24) | <0.001 |
| Anemia | 1.01 (0.87-1.14) | 0.947 |
| Race*age | 1.13 (0.93-1.19) | 0.083 |
| Race*sex | 0.98 (0.82-1.09) | 0.091 |
| Race*diabetes | 1.09 (0.86-1.20) | 0.109 |
| Race*obesity | 1.11 (0.91-1.21) | 0.111 |
| Race*hypertension | 1.05 (0.92-1.24) | 0.092 |
